# Surgeon-led 7-VINCut Antibiotic Stewardship Intervention Decreases Duration of Treatment and Carbapenem Use in a General Surgery Service

**DOI:** 10.3390/antibiotics10010011

**Published:** 2020-12-24

**Authors:** Josep M. Badia, Maria Batlle, Montserrat Juvany, Patricia Ruiz-de León, Maria Sagalés, M Angeles Pulido, Gemma Molist, Jordi Cuquet

**Affiliations:** 1 Department of Surgery, Hospital General Granollers, Universitat Internacional de Catalunya, 08402 Granollers, Spain; mbatlle@fphag.org (M.B.); mjuvany@fphag.org (M.J.); pruizdeleon@fphag.org (P.R.-d.L.); 2 Department of Clinical Pharmacy, Hospital General Granollers, 08402 Granollers, Spain; msagales@fphag.org; 3 Department of Clinical Microbiology, Hospital General Granollers, 08402 Granollers, Spain; apulido@fphag.org; 4 Department of Statistics and Research, Hospital General Granollers, 08402 Granollers, Spain; gmolist@fphag.org; 5 Infectious Diseases Unit, Hospital General Granollers, 08402 Granollers, Spain; jcuquet@fphag.org

**Keywords:** anti-bacterial agents/therapeutic use*, antimicrobial stewardship/organization and administration*, antimicrobial stewardship/statistics and numerical data*, drug resistance, multiple, bacterial/drug effects, infection control/organization and administration, general surgery/standards*, surgical wound infection/prevention and control

## Abstract

Antibiotic stewardship programs optimize the use of antimicrobials to prevent the development of resistance and improve patient outcomes. In this prospective interventional study, a multidisciplinary team led by surgeons implemented a program aimed at shortening the duration of antibiotic treatment <7 days. The impact of the intervention on antibiotic consumption adjusted to bed-days and discharges, and the isolation of multiresistant bacteria (MRB) was also studied. Furthermore, the surgeons were surveyed regarding their beliefs and feelings about the program. Out of 1409 patients, 40.7% received antibiotic therapy. Treatment continued for over 7 days in 21.5% of cases, and, as can be expected, source control was achieved in only 48.8% of these cases. The recommendations were followed in 90.2% of cases, the most frequent being to withdraw the treatment (55.6%). During the first 16 months of the intervention, a sharp decrease in the percentage of extended treatments, with R^2^ = 0.111 was observed. The program was very well accepted by surgeons, and achieved a decrease in both the consumption of carbapenems and in the number of MRB isolations. Multidisciplinary stewardship teams led by surgeons seem to be well received and able to better manage antibiotic prescription in surgery.

## 1. Introduction

According to the World Health Organization (WHO), antimicrobial resistance (AMR) is one of the biggest threats to global health, food security and development [1]. The World Health Assembly in 2015 endorsed a global action plan on AMR, one of its strategic objectives being to optimize the use of antimicrobial medicines [2]. The discovery of antibiotics transformed the practice of medicine in the modern world, making once lethal infections readily treatable and enabling many other medical advances, such as organ transplants and chemotherapy, possible. Early initiation of antibiotics to treat sepsis and severe infections reduces morbidity and mortality [3]. However, the largest driver for the development and spread of AMR is the overuse and misuse of antibiotics [4]. About 30% of all antibiotics prescribed in the Western world in acute care hospitals are either unnecessary or inappropriate [5,6]. Antibiotic Stewardship Programs (ASPs) can help clinicians improve clinical outcomes and minimize harm by improving how antibiotics are prescribed [7]. Optimizing the use of antimicrobials is critical to efficiently fight infections, protect patients from harm, and decrease AMR. Hospital antibiotic stewardship programs can increase infection cure rates while reducing AMR [8], but few of them have been reported as being led by surgeons [9]. It has been argued that surgeons, who actively engage in antimicrobial agent prescription, should play a major role in the establishment and support of ASPs [10].

Carbapenem resistance has dramatically increased worldwide and poses a serious public health threat. In our setting, an increasing high level of carbapenem consumption, mostly related to urinary or intraabdominal infections, has been demonstrated [11], and a duration of 7.6 days in empirical and 11.4 days in directed carbapenem treatment has been observed.

The compliance rates with the suggested duration of therapy in evidenced-based guidelines is low among the surgical community. The use of antibiotics in intra-abdominal infections has been found to be inappropriate in up to 74% of cases, and most of the violations are due to the excessive duration of said therapy [12,13].

The goals of this study were: (1) to decrease the rate of inappropriately prolonged antibiotic treatment in a general surgical service; (2) to assess the effect of the intervention on the consumption of carbapenems and other antibiotics; (3) to assess its effect on the prevalence of AMR in the Department of Surgery; and (4) to assess how the surgeons received the intervention.

## 2. Results

### 2.1. Stewardship Program

Out of 1409 patients evaluated, 573 received antibiotic therapy (40.7%). Of these, 123 underwent treatments for more than 7 days (21.5%), for which a 7-VINCut recommendation was issued. The most important reason that led to a prolonged antibiotic treatment was intraabdominal infection (80.5%). Among these cases, the main diagnostics were community or postoperative peritonitis (38.4%), hepato-biliary infection (36.4%), intraabdominal abscess (15.2%), diverticulitis (5.1%) and pancreatic infection (3.0%). An adequate source control was achieved in only 48.8% of these prolonged treatments, being incomplete or not achieved in the remaining cases.

The antibiotics most frequently targeted by the intervention were piperacillin-tazobactam (37.4%), carbapenems (26.8%), linezolid (13%) and amoxicillin-clavulanate (10.6%).

The issued recommendations are shown in Table 1, and were mainly withdraw or maintain treatment (53.6% and 33.3%, respectively). The recommendations were followed in 111 cases (90.2% adherence).

**Figure 1:**
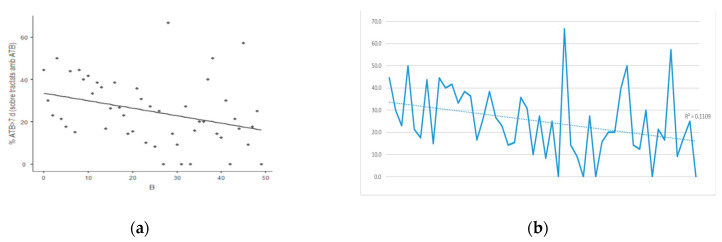
(**a**) Scatterplot (rho = −0.38) and (**b**) Temporal evolution of the percentage of patients on antibiotic treatment with duration over 7 days for the whole study period (R^2^ = 0.111).

There was a significant decrease in treatments >7 days during the overall study period, with R^2^ = 0.111, Spearman rho = −0.38 (IC 95%: −0.6–−0.11; *p* < 0.05) (Figure 1).

Due to the surge of the COVID-19 pandemic in the second part of the Intervention Period (IP), this was split up into two segments of eight months. Comparing these two periods (IP1 and IP2), the hospital stays in the Department of Surgery decreased from 9032 to 5920 (34%), and admissions decreased from 1643 to 1364 (17%), which required an adjustment of the results.

**Figure 2:**
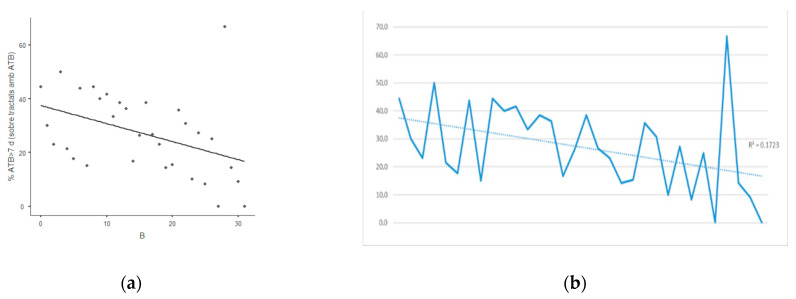
Evolution of the percentage of antibiotic treatments >7 days over patients treated with antibiotics in the Department of Surgery during the first 8 month-period (IP1) (**a)** Scatterplot (rho = −0.492); (**b**) Temporal evolution of the percentage of patients on antibiotic treatment with duration over 7 days (R^2^ = 0.172).

**Figure 3:**
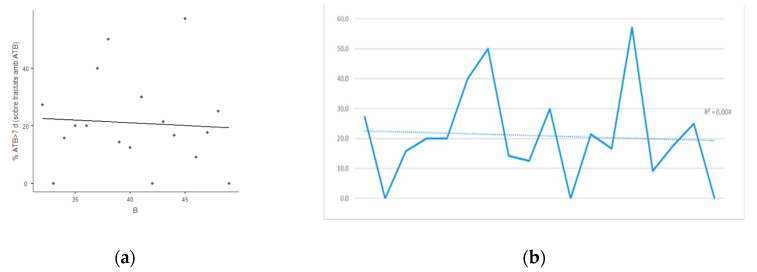
Evolution of the percentage of antibiotic treatments >7 days over patients treated with antibiotics in the Department of Surgery the second 8 month-period, containing the COVID-19 pandemic (IP2). (**a**) Scatterplot (rho = −0.103); (**b**) Temporal evolution of the percentage of patients on antibiotic treatment with a duration of over 7 days (R^2^ = 0.004).

There were 92 prolonged treatments out of 351 patients having antibiotic treatment during the IP1 (26.2%) and 31 out of 222 in IP2 (14.0%). The percentage of prolonged treatments sharply decreased during IP1 (R^2^ = 0.172; Spearman rho = −0.492; 95% CI −0.7–−0.2; *p* < 0.05) (Figure 2), but kept stable during IP2 including the COVID-19 pandemic (R^2^ = 0.004; Spearman rho = −0.103 (95% CI −0.35–−0.11; *p* = 0.68) (Figure 3).

As most of the prolonged treatments came from the surgical emergency department, this flattening of the curve during IP2 could be due to differences in the timing and severity of the emergency cases during the pandemic.

Comparing both periods, no significant differences were observed in the type of diagnoses or the achievement of source control (49.3% vs. 44.2%, *p* = 0.574).

### 2.2. Consumption of Antibiotics

**Figure 4:**
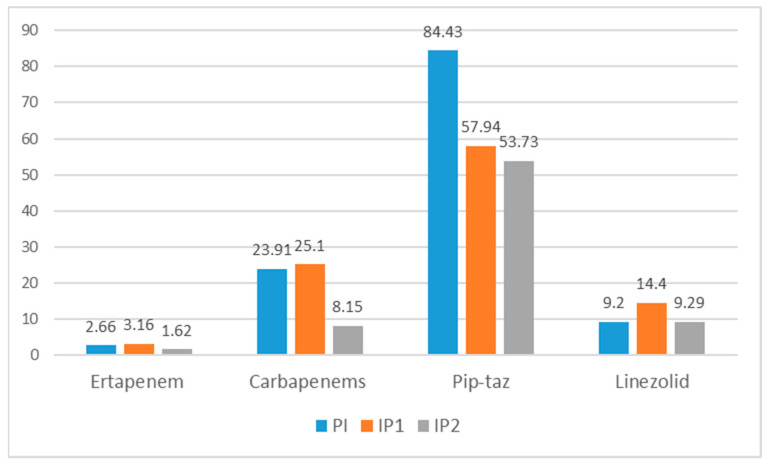
Antibiotic DDD^1^ adjusted per discharges, showing Pre-intervention Period and the two Intervention periods. ^1^ DDD: Defined Daily Dose; PI: Pre-intervention Period; IP1: Intervention Period 1; IP2: Intervention Period 2.

When comparing the period before the intervention (PI) with both intervention periods (IP1 and IP2), the consumption of antibiotics decreased, in particular during IP2, the period covering the final 8 months of the intervention (Figure 4). Table 2 and Table 3 show the individual changes in the use of antibacterials based on DDD/100 stays and DDD/100 discharges.

Comparing the pre-intervention and IP2 periods, the reduction was especially marked for first (ertapenem) and second-line carbapenems (imipenem-cilastatin and meropenem), going from 2.66 to 1.62 DDD/100 discharges and from 0.48 to 0.37 DDD/100 bed-days for ertapenem. The consumption of second-line carbapenems decreased even more, from 23.91 to 8.15 DDD/100 discharges, and from 4.34 to 1.87 DDD/100 bed-days. Regarding second-line carbapenems, there was a relative decrease in imipenem-cilastatin and an increase in meropenem due to changes in the antibiotics policy of the hospital.

### 2.3. Evolution of Multiresistant Bacteria in the Department of Surgery

**Figure 5:**
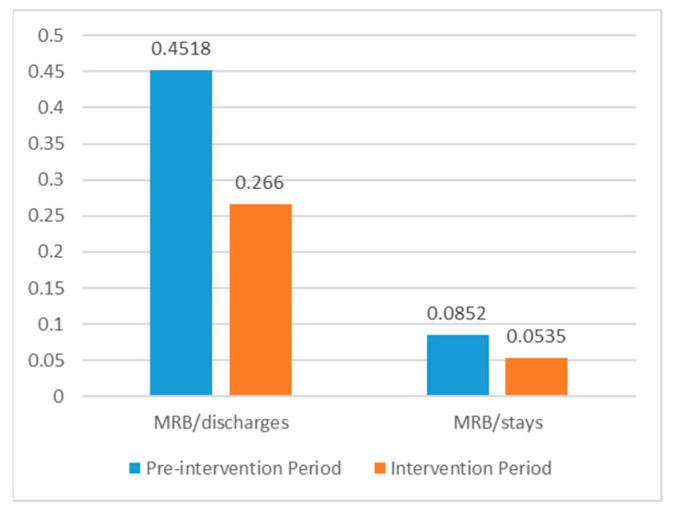
Comparison of the overall incidence of multiresistant bacteria per discharges and bed-days between the Pre-intervention period and the Intervention Period. MR: multiresistant bacteria.

The incidence of multiresistant bacteria (MRB) in the Department of Surgery during the 16 months before and after the intervention was compared. The number of MRB isolations/discharges and MRB isolations/bed-days decreased by 41.1% and 37.2%, respectively, as shown in Figure 5. However, the low number of isolations precluded any statistical analysis.

### 2.4. Internal Evaluation of the 7-VINCut Stewardship Program

The whole surgery department participated in this study, in fact 94.1% of them believed that this program to be a most useful clinical tool. Importantly, over 43% of the surgical team stated that this perception would not change even if other medical doctors rather than surgeons, had participated in the study. However, 56.3% of the respondents considered that the intervention would be less well accepted without the involvement of surgeons in the ASP team.

All those surveyed felt that surgeons should actively participate (64.7%) or lead (35.3%) these stewardship programs in surgery. Regarding the comments found in the electronic medical record, 88.2% of respondents welcomed them, and considered them to be beneficial to their clinical tasks. Only 6% considered the annotations to be an intrusion into their work. The personal percentage of adherence to the recommendations was deemed to be 84.2%. The main reasons for not following the recommendations were disagreement with the comments (35.7%), knowing the patient better (28.6%), and forgetting to make the changes (21.4%).

Respondents considered the most effective ways of improving the program to be educational lectures on antibiotics in surgery, feed-back of stewardship results to providers, and discussion of all recommendations in the teams’ clinical rounds.

## 3. Discussion

Several authors have demonstrated that morbidity is not increased by a shortened course of antibiotics in intraabdominal infection, provided an adequate septic source control is achieved [14,15,16,17,18]. The use of clinical or biological markers such as fever, leukocytosis, or procalcitonin have been described being used to shorten treatment duration [19,20,21]. Other authors found that in patients with complicated intraabdominal infections, a fixed short duration of antibiotic treatment resulted in similar outcomes to those based on resolution of physiological abnormalities [22,23].

Altogether, these data suggest that infectious complications are not improved by a long duration of therapy and that antibiotics may be safely discontinued in a period of time as short as 4 days after an appropriate source control. Longer duration of antibiotic therapy for intraabdominal infection has been even associated with an increased risk of mortality [24].

Current guidelines [25,26,27], recommend no more than 5 to 7 days of antibiotics for complicated intra-abdominal infections depending on the intraoperative findings and resolution of signs and symptoms. They also suggest that patients with continued evidence of infection at the end of the predetermined length of antibiotic therapy (fever, leukocytosis) should be evaluated for an alternative source, likely requiring additional invasive procedures rather than continued antibiotics. If local control is inadequate, a longer duration of therapy might be warranted. Finally, according to these guidelines, completion of an antimicrobial course with oral forms of antimicrobial agents is acceptable in patients able to tolerate an oral diet [26].

In spite of these recommendations, Gorecki et al. found that antibiotics were used inappropriately in 74% of cases of prophylactic and therapeutic administration of antibiotics in surgery. The main reason for inappropriate administration of antibiotics in surgical practice in two-thirds of the cases was excessive duration [12].

Antibiotic Stewardship Programs (ASPs) have been described as “a coherent set of actions designed to use antimicrobials responsibly” [28]. A substantial amount of evidence supports the effectiveness of hospital ASPs [29,30] to optimize antibiotic prescription as well as reduce the incidence of colonization with antibiotic-resistant bacteria and *Clostridium difficile* infections, reduce adverse effects, hospital stay and associated costs [9,31].

ASP teams usually include multidisciplinary experts adequately trained in antibiotic prescribing and stewardship, such as infectious diseases specialists, clinical microbiologists and pharmacists [28]. However, some programs advise including a clinician with expertise in the surgical area whenever possible [32].

It has been argued that surgeons, who actively engage in antimicrobial agent prescription, should play a major role in the establishment and support of ASPs [10]. These stewardship programs are of particular importance to surgical specialties due to their prominent role in prophylactic antibiotic usage and management of surgical infections, but there are few published experiences of ASPs in surgery [9,10,33]. Surgeons should be aware that judicious use of antibiotics is an integral part of any stewardship program and necessary to maximize clinical cure and minimize the emergence of antimicrobial resistance.

### 3.1. Stewardship Program

Less than half of the patients admitted to the surgical wards and included in this study were on antibiotic treatments. Of them, one in five patients had prolonged treatments lasting for over one week. As can be expected, the infections requiring extended treatments were mostly peritonitis or hepato-biliary infections, with an adequate source control achieved in fewer than 50% of cases. Adequacy of source control is crucial to performing short courses of antibiotics in surgical sepsis [22,23,24,25,26,27,28,29,30,31,32,33,34]. Therefore, the low rate of adequate source control in this subset of patients is not surprising.

The main finding of the present study is that a prospective audit combined with a persuasive intervention and feedback to the prescriber can reduce the duration of antibiotic treatments in surgery. It is noteworthy that the results of the intervention were much more successful in the first half of the study than in the second containing the COVID-19 pandemic. This can be due to two reasons. First, the COVID-19 lockdown led to a change in the profile of the patients presenting at the surgical emergency departments (ED). Some of the most frequent emergency surgical conditions showed reductions in their incidence up to 60% (62% for appendicitis, 39% for cholecystitis) [35], probably due to patients’ reluctance to attend hospital [35] or conservative treatments prescribed by primary care physicians [36]. In consequence, the disease seems to have been left to develop at home [35,36], and patients presented with more severe condition [36,37,38], making source control more difficult to achieve. Furthermore, some institutions recommended non-surgical management of cholecystitis or appendicitis [39] placing the emphasis on antibiotic treatment. Moreover, there were changes in the recommendations of the use of some surgical techniques [40], and some authors reported lesser use of the laparoscopic approach [35] (35. Boyle). In summary, patients experienced suboptimal outcomes with more complications and a longer length of stay in the ICU [35] or in-hospital [37], which increased the use of antibiotic treatments.

Second, due to the sudden and extraordinary care load in the hospital during March and April 2020 and the illness of one of the leading team members (with COVID-19), the number of weekly interventions reduced and became irregular. Improvement of the data was slowed, although no worsening was observed. This seems to confirm that continuous stewardship is necessary to maintain good results. At least until such a time as these interventions became a routine part of patient management.

### 3.2. Consumption of Antibiotics

The program also seems to suggest a reduction the consumption of certain antibiotics known to select MR bacteria and the incidence of its isolation, although the study was underpowered to demonstrate those items. However, a reduction of around 50% was observed for both, first and second-line carbapenems, during the period of the study. It should be noted that adherence rates to recommendations related to carbapenem use in ASP interventions have been reported as being not well received [41].

The misuse and overuse of carbapenems, has been associated with the surge of multidrug-resistant or extensively drug-negative Gram-negative bacteria [42]. In surgical departments, there has been a significant increase in the prescription of carbapenems in recent years [11]. Nowadays, Gram-negative rods producing extended spectrum β-lactamases and carbapenemases are the main sources of concern, due to their high speed of dissemination within and between hospitals, and to the risk of them becoming clinically uncontrollable disorders with the associated increased costs [43]. Resistance figures of *Pseudomonas aeruginosa* to meropenem exceeds 20% in the United States and in Spain [44]. In the case of *Klebsiella pneumoniae*, the data have grown considerably in recent years, reaching 4% in Spain, 8% in the United States and up to 36% in Italy in 2015 [44].

### 3.3. Multiresistant Bacteria Isolation

The ultimate goal of an antibiotic stewardship program is to decrease the number of infections produced by mutiresistant bacteria. Although from our data it seems that this has been achieved, we observed a low number of isolations which precluded any statistical analysis. From our present data we are only able to show the variation in the percentage of multiresistant bacteria isolated.

### 3.4. Acceptance of the Stewardship Program

This non-restrictive intervention led by surgeons achieved a good level of approval by providers. Our survey’s results indicate that the high rate of acceptance of ASP recommendations can be related to the presence of a surgeon on this particular ASP team. As acceptance of ASP interventions has been found to be lower among surgeons than by other medical practitioners [41,45], the Global Alliance for Optimizing Rational Antibiotic Use in Intra-Abdominal Infections propose that surgical leaders drive antimicrobial stewardship and education programs to help standardize and improve prescribing behaviors [46]. The engagement of surgeons in antibiotic stewardship programs is likely to be crucial to their success [9,47].

Interestingly enough, some authors found that recommendations to decrease antibiotic exposure, interventions involving carbapenem use, and recommendations to de-escalate or discontinue were associated with lower odds of adherence to ASP interventions [41,45].

Factors that have been associated with increased acceptance included the presence of the ASP physician during rounds, making recommendations verbally [45], and by direct communication via a phone call [41]. In our study, all recommendations were reviewed and discussed weekly at the surgical grand round.

A successful ASP should focus on collaboration among the various professionals within a healthcare institution including prescribing clinicians. In this context, surgeons with knowledge of surgical infections should be directly involved in the improvement projects related to infection in the Departments of Surgery. It is likely that antibiotic guidelines and stewardship programs developed by multidisciplinary teams including surgeons will achieve optimal adherence [48].

However, as it has been shown in the study, the benefits of improvement actions may take some time to be realized, as changes are often difficult to implement in health institutions. According to the Normalization Process Theory [49], patients, professionals, managers, and policy-makers, face two pertinent types of difficulties as they attempt to get advancements into practice: process issues (regarding the utilization of novel perspectives, acting and organizing in health care) and organizational issues (regarding the incorporation of new schemes of practice into existing hierarchical scenarios). Normalization Process Theory is a descriptive model that may help researchers and clinicians understand these procedures, and perhaps may facilitate the introduction of multifaceted processes and new technologies in health systems [50]. Increasing recognition of the failure to translate knowledge into practice has led to greater awareness of the importance of using active dissemination and implementation strategies [51]. Research is still required in this field to explore determining factors of provider behavior to estimate the efficiency of dissemination and implementation strategies in the presence of various barriers and effect modifiers [52]. The ultimate goal of a stewardship should be to stimulate a behavioral change in prescribing practice [46].

### 3.5. Limitations of the Study

This work has several limitations. First, half of the study covered the time of the COVID-19 pandemic, with a concomitant decrease in admissions to surgical units. An attempt has been made to compensate for this by using the DDD per bed-days and per surgical discharge, which in our opinion adjusts data and allows for comparison between both periods. Second, due to the lack of information on variability and dispersion of data, we were unable to statistically demonstrate a change in antibiotic consumption related to the intervention, although we found differences that seem clinically relevant. It is also difficult to calculate accurately its effect on MR bacteria isolation, given its low incidence. These results, although drawn from a single department, may well represent the actual effect of non-restrictive stewardship programs in surgery.

## 4. Materials and Methods

**Figure 6:**
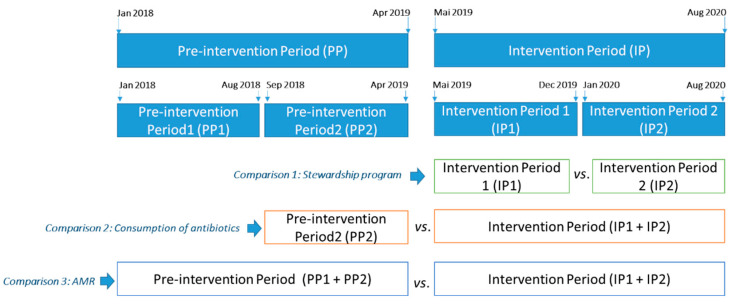
Timeline of the study.

To visualize the effect of the intervention, for some analysis the study period was divided into two 8-month periods: Intervention Period 1 (IP1), from May to December 2019, and Intervention Period 2 (IP2), from January to August 2020, as shown in Figure 6. Given that the COVID-19 pandemic began in the second period, and the admissions and hospital stays in the Department of Surgery were not comparable, some results have been adjusted according to the number of admissions and the hospital stays to allow comparisons.

### 4.1. Stewardship Program

The 7VINCut project started in 2019 at a national level to shorten the duration of antibiotic treatment in surgery. Secondary objectives were to reduce the consumption of carbapenems in surgical services, and to reduce the consumption of other antibiotics with ecological impact (piperacillin-tazobactam, amoxicillin/clavulanate, 3rd and 4th generation cephalosporins and quinolones). 7VINCut promotes the establishment of multidisciplinary teams (surgeon, pharmacologist and infectious diseases specialists) to adapt, in a non-punitive way, antibiotic therapy with the aim of reducing antibiotic treatments to below 7 days in surgical services. Although the intervention focused on the duration of antibiotic treatment, the stewardship team added recommendations on the microbiological appropriateness of the treatments and the use of broad-spectrum antibiotics, especially carbapenems. Before implementation of the program in the hospital, a series of educational initiatives related to antibiotic management were introduced in the Department of Surgery. The prescriptions analyzed were either pathogen directed or empirical broad spectrum antibiotic treatments. Before making the ASP recommendations, the microbiological results were reviewed, when available. The appropriateness of the empirical antibiotics prescribed according to the hospital antibiotic guideline was also reviewed.

The prospective study was conducted between May 2019 and August 2020. All patients hospitalized in the General Surgery Service were prospectively analyzed weekly. A computerized alert allowed the 7VINCut team to identify those patients whose antibiotic treatment lasted longer than 7 days. The team met to individually analyze each of these cases, concluding with a written recommendation in the electronic medical record of each patient. The available recommendations were: withdraw, maintain, de-escalate, broaden, change route, optimize dose, no recommendation. The recommendations were discussed the same day in the General Surgery grand round and implemented if deemed necessary. The 7VINCut program provided educational suggestions for each recommendation made, which were subsequently discussed by the group of surgeons, so it can be considered to be a continuous learning intervention. Adherence to the recommendations was recorded 48 h later by the 7VINCut team.

The main outcome evaluated was the percentage of patients subjected to more than 7 days of antibiotic therapy. To analyze the results of this section, the two Intervention Periods have been used (IP1 and IP2). Variables such as diagnosis of infection, quality of control of infectious focus, use of broad-spectrum antibiotics, route of administration, microbiological adequacy and the percentage of patients subjected to more than 7 days of antibiotic therapy have been analyzed.

### 4.2. Consumption of Antibiotics

Comparative study of antimicrobial use during the eight months before the program implementation (PP2) and during the Intervention Periods (IP1 and IP2). The antimicrobials and routes of administration were selected according to the main objectives of the 7-VINCut program: reduction of the duration of treatments, de-escalation (or spectrum reduction), reduction of carbapenem use, and sequencing of IV to oral therapy. Thus, the considered antimicrobials were: piperacillin-tazobactam (IV), imipenem, meropenem, ertapenem (IV), linezolid (IV and O). The consumption list was obtained through the economic management module of the Pharmacy Service (Farmatools^®^). The consumption in the units that do not generate hospital stays, such as the emergency room, outpatient consultations, day hospital and outpatient dispensing area, were not considered.

The WHO definition of DDD (assumed average maintenance dose per day for a drug used for its main indication in adults), and the WHO classification of DDD of each antimicrobial were used [53]. The DDD per 100 bed-days and DDD per 100 discharges were calculated from the following formula: antimicrobial consumption (grams) during the selected period of time x 100 / DDD x the number of stays (or discharges) during the selected period of time [54]. Consumptions in grams were obtained by multiplying the grams of the pharmaceutical form by the consumption in units. The stays and registrations were provided by the analytical accounting service of the hospital.

A comparison of consumption between DDD/100 bed-days and DDD/100 discharges, was made, according to Collado et al. [55]. This was made because DDD/100 stays reflects the hospital’s exposure to antimicrobials, while DDD/100 discharges better shows changes in hospital activity, which was considered important as the second period of the study included the time of the COVID-19 pandemic.

### 4.3. Multiresistant Bacteria in the Department of Surgery

This section is based in the comparison of the incidence of MRB in the department of surgery during the 16 months before the program implementation (pre-intervention period, PP) and the intervention period (IP). The definition for Gram-positive and Gram-negative bacteria which we used was: bacteria ‘resistant to three or more antimicrobial classes’, according to the definitions of the European Centre for Disease Prevention and Control (ECDC) and the Centers for Disease Control and Prevention (CDC) [56].

The number of MRB was retrieved from the digital records of the microbiology department. Only the organisms considered to be relevant in this surgical department were analyzed (*E. coli, Klebsiella* spp. and *Pseudomonas* spp.). The overall number of MRB per hospital stays and MRB per discharge were used for comparison. Due to the low number of isolations, only the variation in the percentage of multiresistant bacteria isolation is shown.

### 4.4. Internal Evaluation of the 7-VINCut Stewardship Program

At the end of the Intervention Period, a link to a Web-based survey (SurveyMonkey; https://es.surveymonkey.com/r/9GPNTTZ) was circulated via email and WhatsApp to the members of the Department of Surgery. The survey remained open for 20 days. The anonymous questionnaire contained 11 questions aimed at evaluating the beliefs, feelings and suggestions of surgeons about the 7-VINCut intervention.

### 4.5. Ethics and Statistics

The study was approved by the Research Ethics Committee of the Hospital General Universitari de Granollers with code 20202057, which did not consider an informed consent document necessary. The project has been reported according to the “Consolidated criteria for Reporting Qualitative Research (COREQ)” criteria.

Data was entered into a computerized database that was analyzed using the IBM SPSS program (v. 21.0, Chicago, IL, USA). To analyze the relationship between two categorical variables, the chi-square test has been used. Statistical significance was defined at p <0.05. To investigate the temporal evolution of patients on antibiotic treatment with duration over 7 days a linear regression model and a Spearman correlation were used. Since we have worked with aggregated data, the results on antibiotic consumption and MRB isolation have been presented by the percentage of variation in the use of each antibiotic and in the bacteria isolation, not being possible to carry out further statistical comparisons. The results of the survey for the internal evaluation of the program are expressed in percentages of the total answers obtained.

## 5. Conclusions

This 7-VINCut stewardship program achieved a reduction in antibiotic treatment lasting over 7 days. Moreover, this was related with a reduction in the consumption of targeted antibiotics in a General Surgery Service, in particular with carbapenems, and with a reduction in resistant bacteria. Multidisciplinary teams including surgeons seem to be well received by providers, and effective in improving the management of antibiotics in surgical services.

## Figures and Tables

**Table 1:** List of recommendations issued during the stewardship program.

Recommendation	Frequency
Withdraw	66 (53.6%)
Maintain	41 (33.3%)
De-escalate	8 (6.5%)
Broaden	5 (4.1%)
Change route	2 (1.6%)
Optimize dose	1 (0.8%)

**Table 2:** Antibiotic DDD ^1^ adjusted per discharges. Analysis of the variations among Pre-intervention Period and the two Intervention periods.

Antibiotics	PI	IP1	Variation (PI-IP1)	IP2	Variation (IP1-IP2)
Ertapenem	2.66	3.16	↑ 18%	1.62	↓ 48%
Second-line carbapenems	23.91	25.10	↑ 4%	8.16	↓ 67%
Piperacillin-tazobactam	84.43	57.94	↓ 31%	53.73	↓ 7%
Linezolid	9.20	14.40	↑ 56%	9.29	↓ 35%

^1^ DDD: Defined Daily Dose; PI: Pre-intervention Period; IP1: Intervention Period 1; IP2: Intervention Period 2.

**Table 3:** Antibiotic DDD ^1^ adjusted per bed-days. Analysis of the variations among Pre-intervention Period and the two Intervention periods.

Antibiotics	PI	IP1	Variation (PI-IP1)	IP2	Variation (IP1-IP2)
Ertapenem	0.48	0.58	↑ 20%	0.37	↓ 36%
Second-line carbapenems	4.34	4.57	↑ 5%	1.87	↓ 59%
Piperacillin-tazobactam	15.33	10.54	↓ 30%	12.38	↑ 17%
Linezolid	1.67	2.62	↑ 56%	2.14	↓ 18%

^1^ DDD: Defined Daily Dose; PI: Pre-intervention Period; IP1: Intervention Period 1; IP2: Intervention Period 2.

## Data Availability

The data presented in this study are available on request from the corresponding author. The data are not publicly available due to privacy restrictions.
